# Synchronous perforation of a duodenal and gastric ulcer: a case report

**DOI:** 10.1186/1752-1947-4-272

**Published:** 2010-08-18

**Authors:** Dimos Karangelis, Georgios I Tagarakis, Christos Karathanos, Konstantinos Bouliaris, Andony J Baddour, Anargyros Giaglaras

**Affiliations:** 1 Department of General Surgery, General Hospital of Larissa, Greece; 2 Department of Cardiovascular and Thoracic Surgery, University Hospital of Thessaly, Larissa, Greece

## Abstract

**Introduction:**

Peritonitis due to peptic ulcer perforation is a surgical emergency with a high risk of mortality and morbidity.

**Case presentation:**

We present a rare case of a 54-year-old Caucasian man who underwent an emergency laparotomy for peritonitis caused by perforation of two peptic ulcers. The first was located on the anterior wall of the duodenum and the second was posterior, pre-pyloric, close to the lesser curvature.

**Conclusion:**

To the best of our knowledge, this is only the second report in the medical literature of a simultaneous perforation of two peptic ulcers; though rare, every surgeon performing open or laparoscopic repair of a perforated peptic ulcer should be aware of the possibility of simultaneous perforation.

## Introduction

Peptic ulcer disease (PUD; gastric and duodenal ulcers) remains one of the most prevalent and costly gastrointestinal diseases [1]. The annual incidence of peptic ulcer ranges from 0.1% to 0.3% [2]. Internationally, the frequency varies among countries but there are two major precipitating factors: *Helicobacter pylori *infection and the consumption of non-steroidal anti-inflammatory drugs (NSAIDs). Ulcer incidence increases with age for both duodenal ulcers (DUs) and gastric ulcers (GUs) and DUs [3] emerge two decades earlier than GUs, particularly in men. Several factors predict increased risk with NSAIDs, such as *H. pylori *infection, advanced age, comorbidities and adjunct therapy with drugs such as corticosteroids, anticoagulants and bisphoshonates. Complications (bleeding, perforation, obstruction) can occur in patients with peptic ulcers of any etiology. Perforation occurs in about 5% to 10% of patients with active ulcer disease. Duodenal, antral and gastric body ulcers account for 60%, 20% and 20% of perforations, respectively, of peptic ulcers [4,5]. Surgical abdominal exploration (both laparoscopic and laparotomic) is always indicated in gastroduodenal perforation. Hemodynamic instability, signs of peritonitis and free extravasation of contrast material on upper gastrointestinal tract contrast studies make the decision for operation more urgent and imperative. Successful treatment of perforated peptic ulcers with a laparoscopic approach was first reported in 1990 [6,7]. Since then, various institutions have used this technique to treat patients with perforated peptic ulcers. Contraindications for laparoscopic repair for perforated peptic ulcers include large perforations, prior abdominal surgery, a posterior location of the perforation, and a poor general state of health.

## Case presentation

**Figure 1:**
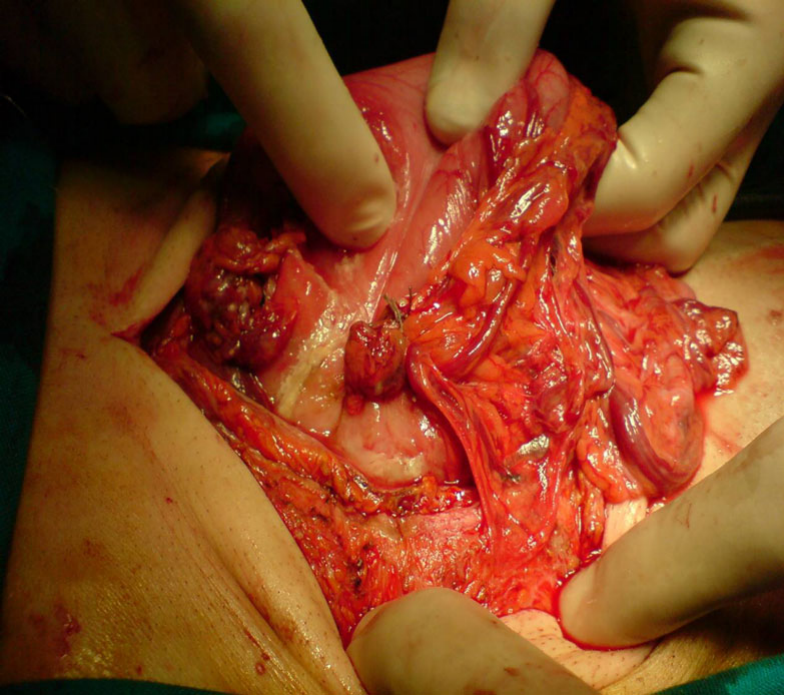
**Omental patch-reinforced suture on the anterior wall of the duodenum**.

**Figure 2:**
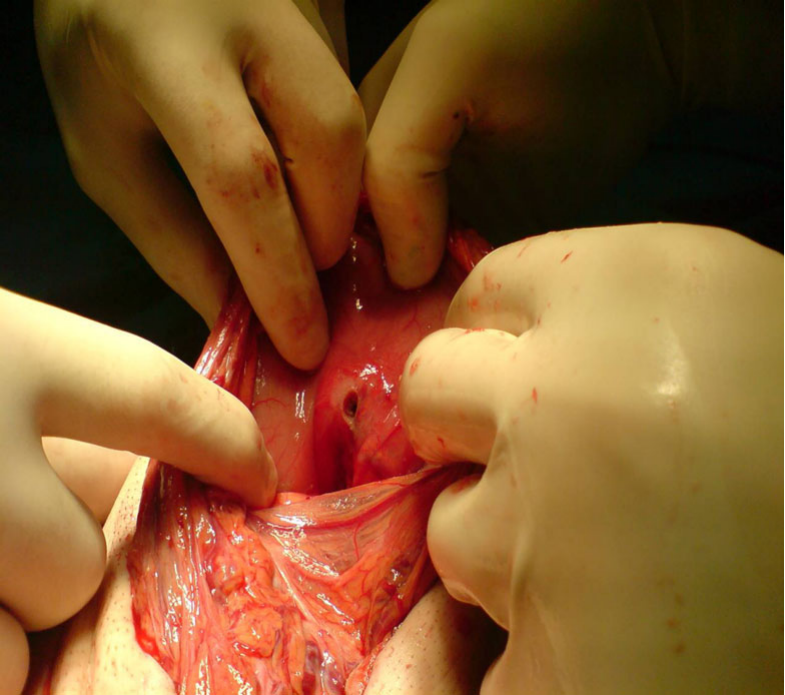
**The second, posterior pre-pyloric ulcer on the lesser curvature of the stomach**.

**Figure 3:**
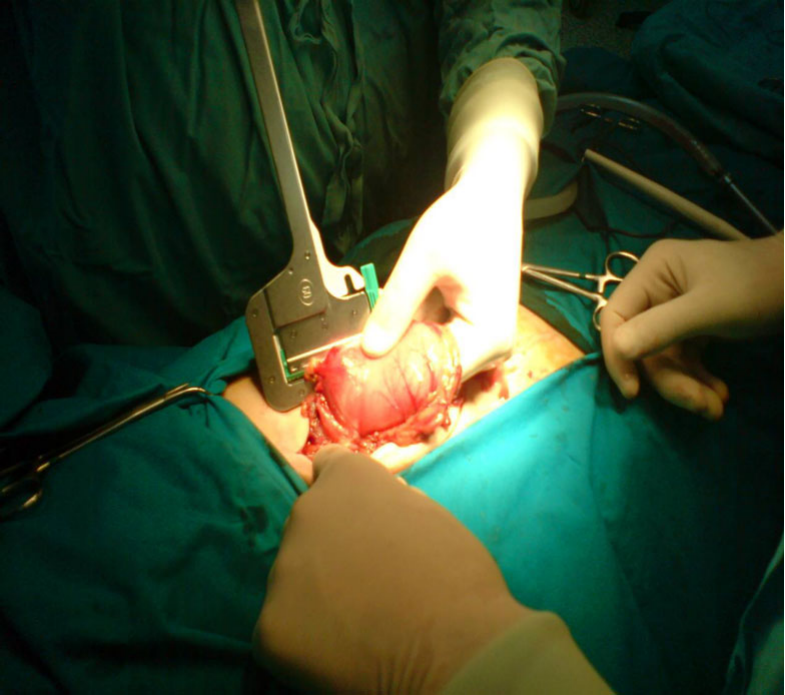
**Wedge resection with staplers**.

A 54-year-old Caucasian Greek man presented to the Accident and Emergency department of our hospital with a 20-day history of abdominal pain, vomiting and loss of appetite. He mentioned an eight kg weight loss over the last 20 days, as he had been drinking almost exclusively water due to his symptoms. He had not presented to any hospital facility earlier because he lived in a remote area in the mountains. On admission, he had the septic image of paleness, tachypnea, tachycardia (110 beats/minute) and a fever of 38.5°C, as well as a rigid abdomen. Abdominal and plain chest X-rays demonstrated free gas under both the hemidiaphragms. After initial resuscitation (placement of intravenous lines and nasogastric tube followed by adequate administration of fluids), our patient underwent an emergency exploratory laparotomy. Our patient's worsening clinical image and his deteriorating clinical signs (tachypnea and tachycardia), along with the presence of his acute abdomen led us to conclude that an emergency laparotomy constituted the treatment of choice. In the face of the emergency situation a computed tomography (CT) scan was not performed. Laparotomy revealed peritonitis due to a perforated ulcer on the anterior wall of the duodenum, which was sutured, while the suture line was reinforced with an omental patch (Figure 1). After a thorough lavage of the peritoneal cavity, further exploration of the intra-abdominal organs revealed a second posterior pre-pyloric ulcer on the lesser curvature of the stomach, perforated into the lesser sac (Figure 2). A wedge resection with staplers was carried out (Figure 3), while no further acid reduction procedures were undertaken due to sepsis. A Nissen fundoplication was performed as an anti-reflux measure. Our patient recovered uneventfully and was discharged home on the 13th post-operative day; at this time we administered an appropriate eradication therapy. More specifically, we followed the protocol of triple therapy: a proton pump inhibitor, amoxicillin and clarithromycin were administered. After discharge our patient was referred to gastrointestinal specialists. Our colleagues planned a surveillance endoscopy according to their protocol.

## Discussion

Perforation of a peptic ulcer is a surgical emergency that still carries a risk of mortality. We successfully managed a rare and difficult case of simultaneous perforation of duodenal and gastric ulcers that could have been easily misdiagnosed and undertreated. Retrospectively studying our case, we can state that there is a growing experience with laparoscopic techniques for management of peptic ulcers. A Graham patch, with or without a laparoscopic vagotomy for perforated peptic ulcers is probably the most appropriate minimally invasive approach when in experienced hands [8,9]. Nevertheless, this case raises doubts as to the extent laparoscopy would have been a safe procedure in our case in terms of revealing both lesions.

Finally, every surgeon should strictly follow one of the basic principles of abdominal surgery and perform a thorough examination of the peritoneal cavity in every case of diffuse peritonitis, even if the underlying pathology appears to be obvious.

## Conclusions

In summary, emergency physicians and surgeons should maintain a high level of clinical suspicion as a second perforative peptic lesion, though a rare possibility, could exist and could potentially be lethal.

## Competing interests

The authors declare that they have no competing interests.

## Authors' contributions

KD was the primary surgeon for the case, conducted a thorough literature research, and was the chief author in terms of writing the paper. TG performed a consultation regarding the anti-reflux treatment, and co-authored the paper. KC assisted with the linguistics and performed literature research. BK assisted with the literature research. BA performed literature research and checked the final version of the manuscript. GA was the attending surgeon for the case and checked the paper. All authors read and approved the final manuscript.

## Consent statement

Written informed consent was obtained from the patient for publication of this case report and accompanying images. A copy of the written consent is available for review by the Editor-in-Chief of this journal.
